# Modulation of the vitamin D/vitamin D receptor system in osteoporosis pathogenesis: insights and therapeutic approaches

**DOI:** 10.1186/s13018-023-04320-4

**Published:** 2023-11-13

**Authors:** Yanqi Li, Pengfei Zhao, Biyun Jiang, Kangyong Liu, Lei Zhang, Haotian Wang, Yansheng Tian, Kun Li, Guoqi Liu

**Affiliations:** 1 Central Laboratory, Huabei Petroleum Administration Bureau General Hospital, Huidaozhan Avenue, Renqiu City, 062552 Hebei Province China; 2 Biotecnovo (Beijing) Co. Ltd., Building 12, Yard 20, Guangde Street, Beijing Economic and Technological Development Zone, Beijing, 100176 China; 3 Clinical School of Medicine, North China University of Science and Technology, Tangshan, 063000 Hebei China; 4 No.1 Department of Orthopedics, Langfang People’s Hospital, No 37, Xinhua Rd, Langfang, 065000 Heibei China

**Keywords:** Osteoporosis, Vitamin D receptor, Bone mineral density, Bone remodeling, Calcium metabolism, Genetic variations

## Abstract

Osteoporosis is a prevalent bone disorder characterized by low bone mineral density (BMD) and deteriorated bone microarchitecture, leading to an increased risk of fractures. Vitamin D (VD), an essential nutrient for skeletal health, plays a vital role in maintaining bone homeostasis. The biological effects of VD are primarily mediated through the vitamin D receptor (VDR), a nuclear receptor that regulates the transcription of target genes involved in calcium and phosphate metabolism, bone mineralization, and bone remodeling. In this review article, we conduct a thorough literature search of the PubMed and EMBASE databases, spanning from January 2000 to September 2023. Utilizing the keywords “vitamin D,” “vitamin D receptor,” “osteoporosis,” and “therapy,” we aim to provide an exhaustive overview of the role of the VD/VDR system in osteoporosis pathogenesis, highlighting the most recent findings in this field. We explore the molecular mechanisms underlying VDR’s effects on bone cells, including osteoblasts and osteoclasts, and discuss the impact of VDR polymorphisms on BMD and fracture risk. Additionally, we examine the interplay between VDR and other factors, such as hormonal regulation, genetic variants, and epigenetic modifications, that contribute to osteoporosis susceptibility. The therapeutic implications of targeting the VDR pathway for osteoporosis management are also discussed. By bringing together these diverse aspects, this review enhances our understanding of the VD/VDR system’s critical role in the pathogenesis of osteoporosis and highlights its significance as a potential therapeutic target.

## Introduction

Osteoporosis is a significant and growing health concern worldwide, particularly among the aging population [1, 2]. It is characterized by reduced bone mineral density (BMD) and deteriorated bone microarchitecture, resulting in increased skeletal fragility and a heightened risk of fractures [1, 2]. According to the International Osteoporosis Foundation, osteoporosis affects an estimated 200 million individuals globally, and this number is projected to rise as the population continues to age [3]. The overall prevalence of osteoporosis among adults aged 50 and over is nearly 13% while it rises to approximately 49% among women over 50 ages [2, 3]. The present rapid growth of the elderly population has made osteoporosis a major healthcare burden that has resulted in enormous and growing costs to national healthcare systems [1–3].

**Fig. 1:**
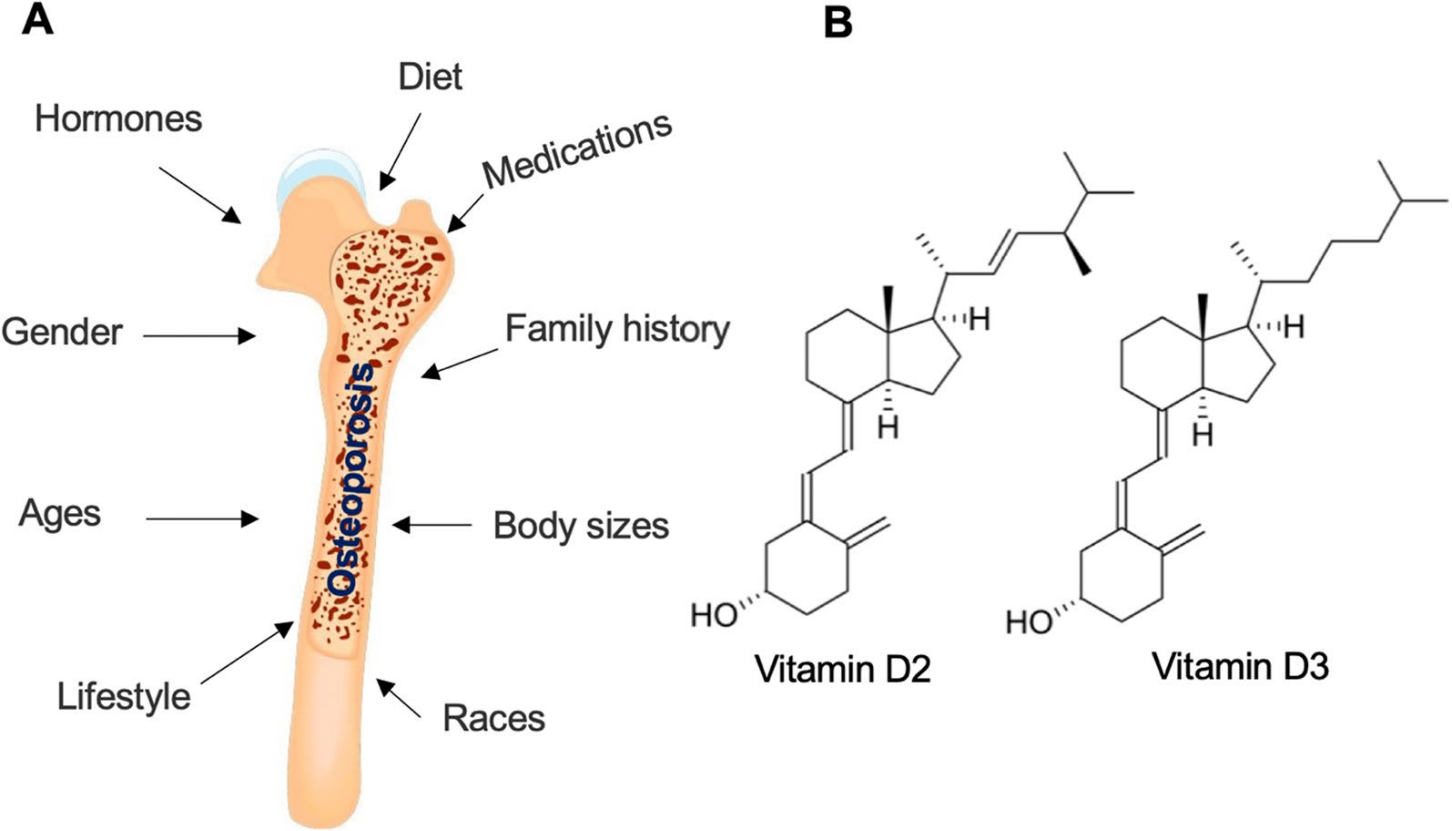
Factors involved in the pathogenesis of osteoporosis and chemical structures of vitamin D2/D3. **A** Factors contributing to pathogenesis of osteoporosis. Osteoporosis can be affected by multiple factors, such as gender, age, race, body size, family history, diet, changes in hormones, lifestyle, and special medicines. **B** Chemical structures of vitamin D2 (C28H44O) and D3 (C27H44O) are shown

The pathogenesis of osteoporosis is complex and multifactorial, involving a delicate interplay between genetic, hormonal, environmental, and lifestyle factors (Fig. 1A) [4, 5]. Importantly, a deficiency of vitamin D (VD) is an important cause that leads to osteoporosis [6]. It is crucial to understand the underlying mechanisms that contribute to bone loss and compromised bone quality in order to develop effective preventive and therapeutic strategies.

VD, in particular VD2 and VD3 (Fig. 1B), is a group of fat-soluble secosteroids vital for skeletal health, and it plays a key role in maintaining bone homeostasis [7, 8]. Its biological effects are primarily mediated via the vitamin D receptor (VDR), a nuclear receptor that governs the transcription of target genes [7, 8]. These genes are involved in a wide array of processes, including calcium and phosphate metabolism, bone mineralization, and bone remodeling, all critical to the preservation of bone health [9]. Dysregulation of the VD/VDR system has been linked to several bone pathologies, including osteoporosis [10]. Recent advancements have shed light on how the VD/VDR system influences the behavior of bone cells, such as osteoblasts and osteoclasts, and how VDR polymorphisms might affect BMD and fracture risk [1, 11, 12]. Moreover, the interplay between the VDR and other factors, such as hormonal regulation, genetic variants, and epigenetic modifications, has become a topic of intense investigation [13, 14].

In this review, we aim to provide a comprehensive overview of the current understanding of the VD/VDR system's role in osteoporosis pathogenesis. By consolidating recent findings, we underscore the relevance of the VD/VDR system in the onset and progression of osteoporosis, while also highlighting potential therapeutic avenues for disease management.

## Uptake, synthesis, and metabolism of VD

**Fig. 2:**
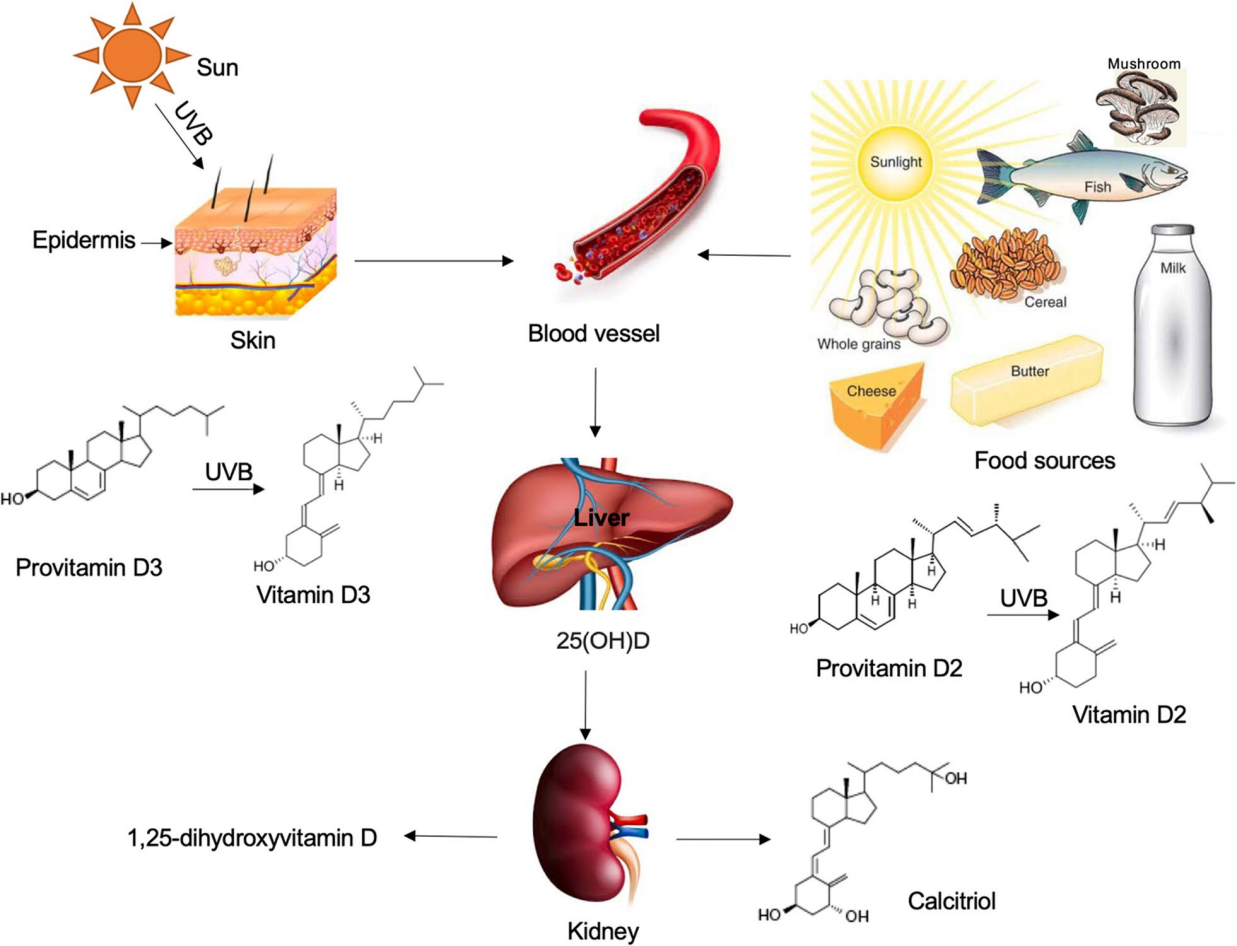
Uptake, synthesis, and metabolism of vitamin D2 and D3. Vitamin D can be obtained through natural sun exposure and from dietary sources such as fish, mushrooms, cheeses, milk, butter, cereal, and whole grains. Vitamin D3 is synthesized in the skin when pro-vitamin D3 (7-dehydrocholesterol) is converted to pre-vitamin D3 in response to sunlight exposure (ultraviolet B radiation). Alternatively, Vitamin D3 can be obtained from natural or fortified foods and supplements from vitamin D2 (ergocalciferol) and vitamin D3. After entering the bloodstream, vitamin D2 and D3 bind to vitamin D-binding protein (DBP) and are transported to the liver where they are hydroxylated by liver 25-hydroxylases to form 25-hydroxycholecalciferol [25(OH)D] (calcifediol or calcidiol). 25(OH)D is then transported to the kidney where it is further hydroxylated by 1α-hydroxylase to produce the active secosteroid 1,25(OH)2D (calcitriol). The synthesis of 1,25(OH)2D is regulated by parathyroid hormone and is suppressed by calcium, phosphate, and 1,25(OH)2D itself

VD can be sourced from natural sun exposure and certain foods, including fatty fish, fish liver oils, beef liver, egg yolks, and mushrooms. Additionally, various foods, such as cheeses, milk, butter, cereal, and whole grains are often fortified with VD supplements to enhance dietary intake (Fig. 2) [7, 15]. The synthesis of VD begins in the skin, where exposure to ultraviolet B (UVB) radiation converts 7-dehydrocholesterol, a compound found in significant amounts in the skin, into previtamin D3 (Fig. 2) [7, 15]. Previtamin D3 is then transformed into vitamin D3 (VD3), or cholecalciferol, via a heat-dependent process (Fig. 2) [7, 15]. Following its synthesis, VD3 is metabolized in the liver by the enzyme 25-hydroxylase to 25-hydroxyvitamin D3, also known as calcifediol (Fig. 2) [7, 15].

VD metabolism occurs primarily in the kidneys but also in other tissues, where the enzyme 1α-hydroxylase converts calcifediol into its active form, calcitriol (1,25-dihydroxyvitamin D3) (Fig. 2) [7, 15, 16]. This conversion is tightly regulated by several factors including serum levels of parathyroid hormone, calcium, and phosphate [11]. Recent studies have found that dysregulation in this final conversion step could lead to inadequate levels of active VD, contributing to the pathogenesis of various health conditions including osteoporosis [17].

In addition to endogenous synthesis, dietary intake also contributes to the body's VD status [7, 8]. The primary dietary forms of VD are vitamin D3, found in foods like fatty fish and egg yolks, and vitamin D2, or ergocalciferol, which is obtained from plant sources and fortified foods (Fig. 2) [7, 8]. Both forms are metabolized in the same manner within the body [7, 8].

## Structure and functions of VDR

### Molecular structure of VDR

**Fig. 3:**
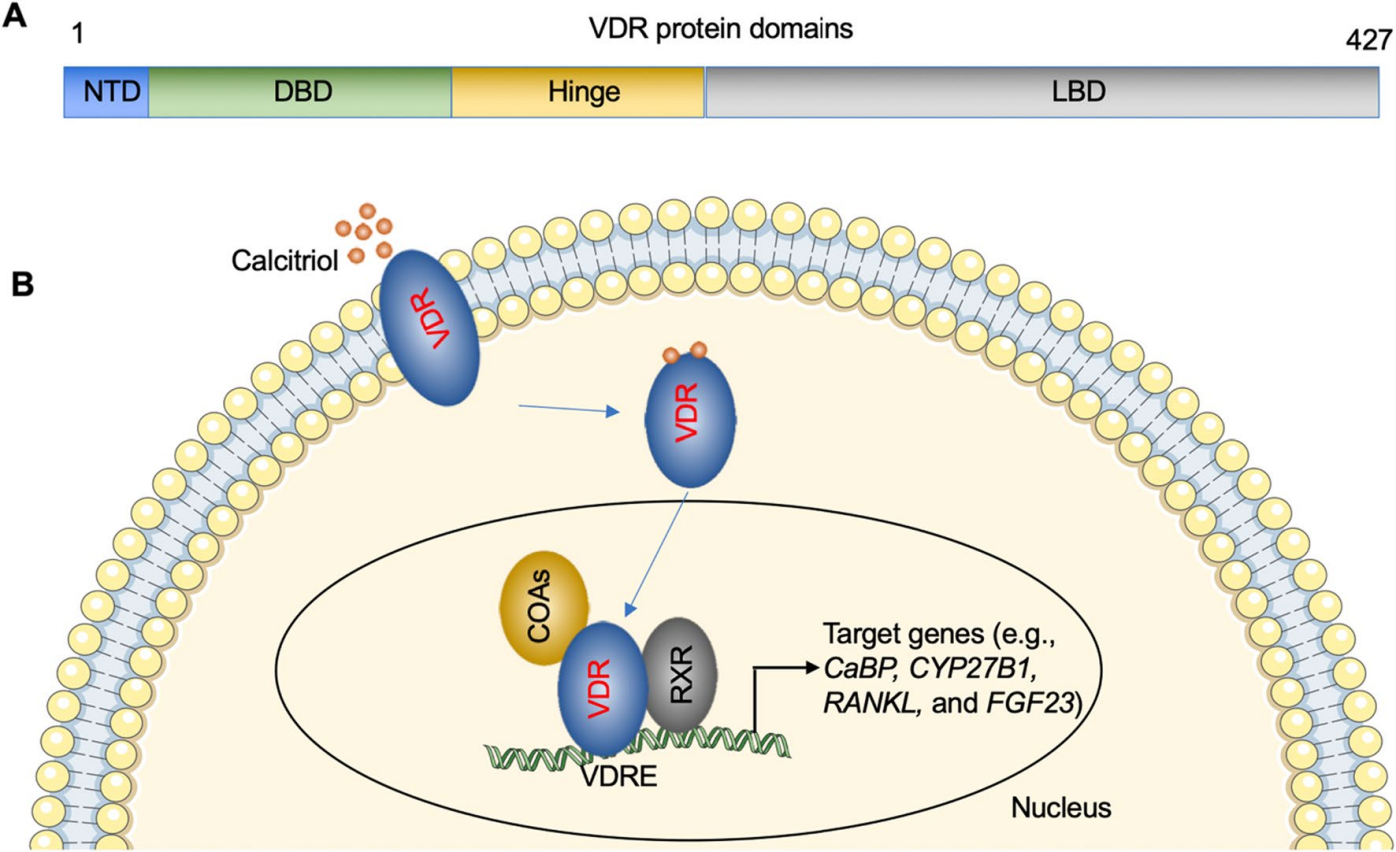
Functional domains of VDR protein and activation of VDR. **A** Functional domains of VDR proteins. Different domains, including NTD, DBD, and LBD, are shown. **B** The classic VDR signaling. The VDR-ligand complex forms a heterodimer with RXR, and the complex translocates to the nucleus. In the nucleus, the VDR-RXR heterodimer binds to VDREs located in the promoter regions of target genes, leading to the recruitment of coactivators and the initiation of gene transcription

VDR is a nuclear hormone receptor that regulates gene expression in response to VD [18]. The VDR protein consists of several domains that contribute to its structure and function [19, 20]. The N-terminal domain (NTD) contains transcriptional activation functions and interacts with coactivators and corepressors (Fig. 3A) [19, 20]. The DNA-binding domain (DBD) allows the VDR to bind to specific DNA sequences called vitamin D response elements (VDREs) (Fig. 3A) [19, 20]. The ligand-binding domain (LBD) is responsible for ligand recognition and dimerization with the retinoid X receptor (RXR) (Fig. 3A) [19, 20]. Together, these domains enable the VDR to interact with DNA and other proteins to modulate gene expression [19, 20].

### Ligand binding and activation of VDR

VDR is activated by binding to its ligand, calcitriol [21]. Upon ligand binding, the VDR undergoes conformational changes that facilitate its interaction with coactivator proteins [22]. The VDR-ligand complex then forms a heterodimer with RXR, and the complex translocates to the nucleus [22]. In the nucleus, the VDR-RXR heterodimer binds to VDREs located in the promoter regions of target genes, leading to the recruitment of coactivators and the initiation of gene transcription (Fig. 3B) [22]. The ligand-binding process is crucial for the activation of VDR and the regulation of target gene expression [22].

### VDR signaling pathways

Activation of VDR can trigger a multitude of signaling pathways that contribute to its myriad physiological effects, ranging from maintaining bone homeostasis to modulating immune responses [23–25]. The binding of VDR–RXR heterodimer to VDREs on gene promoters represents the classic genomic pathway of VDR, which has been shown to regulate numerous genes [e.g., Calbindin (CaBP), Cytochrome P450, Family 27, Subfamily B, Polypeptide 1 (CYP27B1), nuclear factor kappa-Β ligand (RANKL), fibroblast growth factor 23 (FGF23)] involved in calcium and phosphate metabolism (Fig. 3B), crucial for bone health [9, 23, 26].

**Fig. 4:**
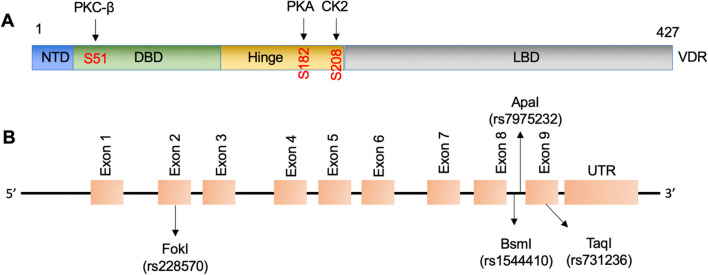
Phosphorylation sites of VDR by kinases and chromosomal positions of VDR polymorphisms. **A** Phosphorylation sites of VDR by kinases (PKC-β, PKA, and CK2). **B** Chromosomal positions of four VDR polymorphisms (rs7975232, rs1544410, rs228570, and rs731236) are shown

Besides the classic pathway, VDR can also initiate rapid non-genomic actions, which take effect within minutes to hours of VD administration [27, 28]. In these pathways, VDR interacts with and is phosphorylated by various protein kinases, such as protein kinase A (PKA), mitogen-activated protein kinase (MAPK), protein kinase C beta (PKC-β), and casein kinase 2 (CK2) or with phospholipase C to modulate intracellular calcium and phosphate levels (Fig. 4A) [27, 29, 30].

Moreover, VDR can interact with other signaling molecules, exemplified by its crosstalk with the Wnt/β-catenin signaling pathway, which is instrumental for bone formation [31, 32]. In addition, VDR has been found to interact with other nuclear receptors, such as estrogen receptor (ER) and peroxisome proliferator-activated receptor gamma (PPARγ), indicating complex interactions in regulating bone health and inflammation [33].

Emerging evidence indicates a pivotal role for the VD/VDR signaling pathway in modulating inflammatory responses as well. Some studies have demonstrated the inhibitory effect of VD/VDR signaling on the activation of the NF-κB pathway, a key player in inflammation, in specific cell types such as embryonic fibroblasts and intestinal epithelial cells [34–37]. Moreover, VD has been found to downregulate the expression of hypoxia-inducible factor-1α (HIF-1α) in osteoclasts, while tumor necrosis factor-alpha (TNFα) appears to reciprocally decrease VDR levels [38]. Additionally, VDR signaling has been implicated in immune regulation, cell proliferation, and differentiation [39, 40]. The VDR is expressed in immune cells, and its activation can modulate immune responses, such as the production of antimicrobial peptides and the regulation of immune cell differentiation [39, 40].

### Regulation of VDR expression

The expression of VDR can be regulated at multiple levels. Transcriptional regulation plays a crucial role in determining VDR abundance. Factors such as VD status, calcium levels, and hormonal signals can influence VDR gene transcription [25, 41]. Additionally, posttranscriptional mechanisms, including mRNA stability and microRNA-mediated regulation, can impact VDR expression [25, 41]. Furthermore, epigenetic modifications, such as DNA methylation and histone modifications, can affect VDR expression by altering chromatin structure and accessibility [25, 41]. These modifications can be influenced by environmental factors, including diet, lifestyle, and exposure to sunlight [25, 41].

## Effects of VD/VDR on osteoblasts and osteoclasts

### VDR regulation of osteoblast function and mineralization

Osteoblasts, the cells responsible for bone formation, play a crucial role in maintaining skeletal integrity and bone remodeling [42]. Emerging evidence has highlighted the importance of VDR in regulating osteoblast function and differentiation [43, 44]. After binding of calcitriol, VDR activation promotes osteoblast differentiation, a tightly regulated process involving the transition of mesenchymal stem cells into mature osteoblasts [43, 44]. Studies have demonstrated that VDR activation enhances the expression of key osteogenic markers, such as runt-related transcription factor 2 (Runx2), osterix (Osx), and alkaline phosphatase (ALP) [45–47]. These factors are essential for osteoblast commitment and maturation, as well as the subsequent synthesis and mineralization of the bone matrix [48].

Beyond its role in osteoblast differentiation, VDR exerts profound effects on osteoblast function and mineralization [49]. VDR activation stimulates the production of various extracellular matrix proteins, including type I collagen, osteopontin, and osteocalcin, which are essential for bone formation and maintenance of bone strength [43, 44]. Moreover, VDR activation modulates the balance between osteoblast-mediated bone formation and osteoclast-mediated bone resorption by influencing the expression of receptor activator of nuclear factor kappa-B ligand (RANKL) and osteoprotegerin (OPG) [50–52]. RANKL is a key osteoclastogenic factor, while OPG acts as a decoy receptor, inhibiting RANKL and preventing osteoclast activation [50–52]. VDR activation in osteoblasts leads to decreased RANKL expression and increased OPG expression, thereby suppressing osteoclastogenesis and maintaining bone homeostasis [50–52].

### VDR regulation of osteoclast function and bone resorption

Osteoclasts, the cells responsible for bone resorption, play a crucial role in maintaining bone homeostasis through the removal of old or damaged bone tissue [53]. Emerging evidence suggests that VDR is involved in the regulation of osteoclast differentiation and activation. Studies have demonstrated that VDR activation inhibits osteoclast differentiation by suppressing the expression of key osteoclastogenic factors such as RANKL and macrophage colony-stimulating factor (M-CSF) [54]. VDR activation reduces the production of RANKL and M-CSF, thereby inhibiting osteoclast formation and subsequent bone resorption [54].

In addition to its effects on osteoclast differentiation, VDR has been shown to regulate the function of mature osteoclasts and the process of bone resorption [54, 55]. VDR activation has been reported to suppress the expression of genes involved in osteoclast activity, such as cathepsin K, matrix metalloproteinase-9 (MMP-9), and tartrate-resistant acid phosphatase (TRAP) [56]. Cathepsin K is a lysosomal protease involved in the degradation of the organic matrix of bone, while MMP-9 participates in the breakdown of the collagenous matrix [56]. TRAP is an enzyme associated with osteoclast function and is used as a marker for osteoclast activity [56]. By downregulating the expression of these genes, VDR activation reduces the resorptive capacity of osteoclasts [56]. By increasing the OPG-to-RANKL ratio and Wnt/β-catenin signaling, VDR activation helps maintain a balance between bone formation and resorption [32].

## Genetic variations in the VDR gene and osteoporosis

### Polymorphisms in the VDR gene and osteoporosis susceptibility

The human *VDR* gene is located on the short arm of chromosome 12q13.1 [57]. This gene is more than 100 kb in length and consists of 6 untranslated exons (exons 1a–1f) and 8 protein-coding exons (exons 2–9) [58]. More than 60 polymorphisms of the *VDR* gene have been reported, ranging from sites in the promoter, exons, and introns to the 3’-untranslated region (UTR) [59]. Genetic variations in the VDR gene have been extensively studied in relation to osteoporosis susceptibility. Several single nucleotide polymorphisms (SNPs) have been identified in the VDR gene, and these variations can influence VDR function and subsequent effects on bone health. Commonly studied VDR SNPs include FokI (rs2228570), ApaI (rs7975232), BsmI (rs1544410), and TaqI (rs731236). Of these polymorphisms, ApaI and BsmI are located in the intron between exon 8 and exon 9, FokI is present in exon 2, and TaqI is located in exon 9 (Fig. 4B) [60].

Extensive research has been carried out globally on the relationship between VDR gene polymorphisms, particularly ApaI, TaqI, and BsmI, and osteoporosis (Table 1) [61–75]. Although there are variations in study results, a significant association between these polymorphisms and osteoporosis risk has been predominantly reported (Table 1). Notably, studies conducted in various ethnic populations including Saudis, British, and Chinese revealed significant associations between these polymorphisms and BMD or osteoporosis risk, despite some discrepancies (Table 1) [61–75].

**Table 1:** Association of different VDR polymorphisms with osteoporosis risks

Polymorphism	Osteoporosis Risk	Ethnicity	References
ApaI TaqI	Significantly associated with osteoporosis incidence risk	Saudi, White British males, Postmenopausal Chinese women, Korea Saudi Arabia	[61, 62, 66, 67, 69]
ApaI TaqI	No correlation with BMD	Austria, Spanish women aged over 60	[64, 71]
BsmI	Significant association with osteoporosis susceptibility	Caucasians, Asian	[65, 70]
BsmI	No statistical association with osteoporosis risk and BMD	Postmenopausal Spanish women undergoing osteoporosis treatment, Han Chinese	[71, 74]
FokI	Potential biomarker for osteoporosis development	Postmenopausal Thai, Italian postmenopausal women, Chinese, Caucasian	[63, 68, 72, 75]
FokI	Association with osteoporosis risk in Asians, not in Caucasians	Meta-analysis	[73]
rs11568820	Association with risk of fracture and osteoporosis	Young, healthy, postmenopausal Spanish women	[76]
rs11568820	No significant association with osteoporosis risk	Meta-analysis	[12]
p.Gly14Ala p.His305Gln	Association with osteoporosis risk	Postmenopausal Chinese women	[77]

In addition to the mentioned polymorphisms, others like rs11568820, and genetic variants p.Gly14Ala and p.His305Gln have been identified as potential osteoporosis predictors in certain populations (Table 1) [12, 76, 77]. Nevertheless, their association with osteoporosis risk remains inconclusive, as results varied among different studies.

### Influence of VDR polymorphisms on BMD

BMD is a key indicator of bone health and osteoporosis risk [2]. Multiple studies have reported that the FokI, BsmI, TaqI, and ApaI polymorphisms significantly impact BMD in different populations, thereby influencing osteoporosis risk. However, these findings have been inconsistent, with some studies revealing significant associations between these polymorphisms and BMD, while others show no such relationship [61–75]. This disparity has been attributed to factors such as ethnic diversity and genetic heterogeneity.

Recent advances in genomic research have further shed light on how VDR polymorphisms might interact with environmental factors to influence BMD. For instance, the impact of VDR polymorphisms on BMD may be modulated by VD levels, dietary calcium intake, and sun exposure [78, 79]. Several studies have reported that the association between VDR polymorphisms and BMD becomes more pronounced under conditions of low calcium intake or VD deficiency [80, 81]. Additionally, research has started to unravel the mechanisms through which these polymorphisms might influence BMD, with data suggesting that they can affect the efficiency of VD signaling, and therefore the regulation of calcium and phosphate homeostasis [79–81]. However, further research is needed to fully understand the complex interactions between VDR polymorphisms, BMD, and osteoporosis risk.

## Interplay between VDR and other key players in bone metabolism

### VDR and parathyroid hormone

The interplay between VDR and parathyroid hormone (PTH) is crucial for maintaining calcium homeostasis and bone health [82]. PTH plays a central role in regulating serum calcium levels, while VD and its receptor are involved in calcium absorption and utilization in the body [82]. The VDR is expressed in the parathyroid glands, where it modulates the production and secretion of PTH [83]. PTH acts on target tissues, such as the kidneys and bones, to increase calcium levels [83]. Through its interaction with the VDR, PTH regulates the expression of genes involved in calcium metabolism and bone remodeling [18, 82, 83].

### VDR and calcium-sensing receptor

The calcium-sensing receptor (CaSR) is another key player in calcium homeostasis [84]. It is expressed in various tissues, including the parathyroid glands, kidneys, and bones [84]. The CaSR detects changes in extracellular calcium levels and regulates PTH secretion accordingly [85]. The VDR and CaSR have a complex interplay in maintaining calcium balance. VD, acting through the VDR, stimulates intestinal calcium absorption, which indirectly affects the activity of the CaSR [84, 85]. The CaSR, in turn, modulates PTH secretion and calcium reabsorption in the kidneys [84, 85]. Both the VDR and CaSR are involved in feedback mechanisms that regulate PTH production and calcium levels in the body [84, 85].

### VDR and estrogen receptor

Estrogen plays a critical role in bone health, and its deficiency contributes to the development of osteoporosis [86]. The estrogen receptor (ER) and VDR have interactions that influence bone metabolism [87, 88]. Estrogen stimulates calcium absorption in the intestines and inhibits bone resorption by osteoclasts [89]. Estrogen and VD signaling pathways can cross-regulate each other, highlighting the interconnectedness between these receptors in maintaining bone health.

Understanding the interplay between the VDR, PTH, CaSR, and ER is crucial for unraveling the complex mechanisms involved in bone metabolism and calcium homeostasis. Dysregulation or disruption of these interactions can lead to imbalances in bone remodeling, impaired calcium absorption, and increased osteoporosis risk. Further research is needed to elucidate the intricate molecular pathways and regulatory mechanisms underlying the interplay between the VDR and other key players in bone metabolism. This knowledge may open new avenues for targeted therapeutic interventions for bone-related disorders, including osteoporosis.

## Therapeutic implications of targeting the VD/VDR signaling

### VD supplementation in osteoporosis

VD supplementation is a widely used therapeutic approach in the management of osteoporosis [90, 91]. As VD is essential for calcium absorption and bone mineralization, inadequate levels can contribute to bone loss and increased fracture risk [90, 91]. Supplementation with VD aims to optimize serum VD levels and enhance calcium absorption, thereby improving bone health [90, 91].

Clinical studies have shown that VD supplementation, especially when combined with calcium, can reduce the risk of fractures in osteoporotic individuals [90, 91]. The optimal dosage and duration of VD supplementation vary depending on individual factors such as age, sunlight exposure, and baseline VD levels [90, 91]. Regular monitoring of serum VD levels is recommended to ensure adequate supplementation [90, 91].

### Selective VDR modulators

**Fig. 5:**
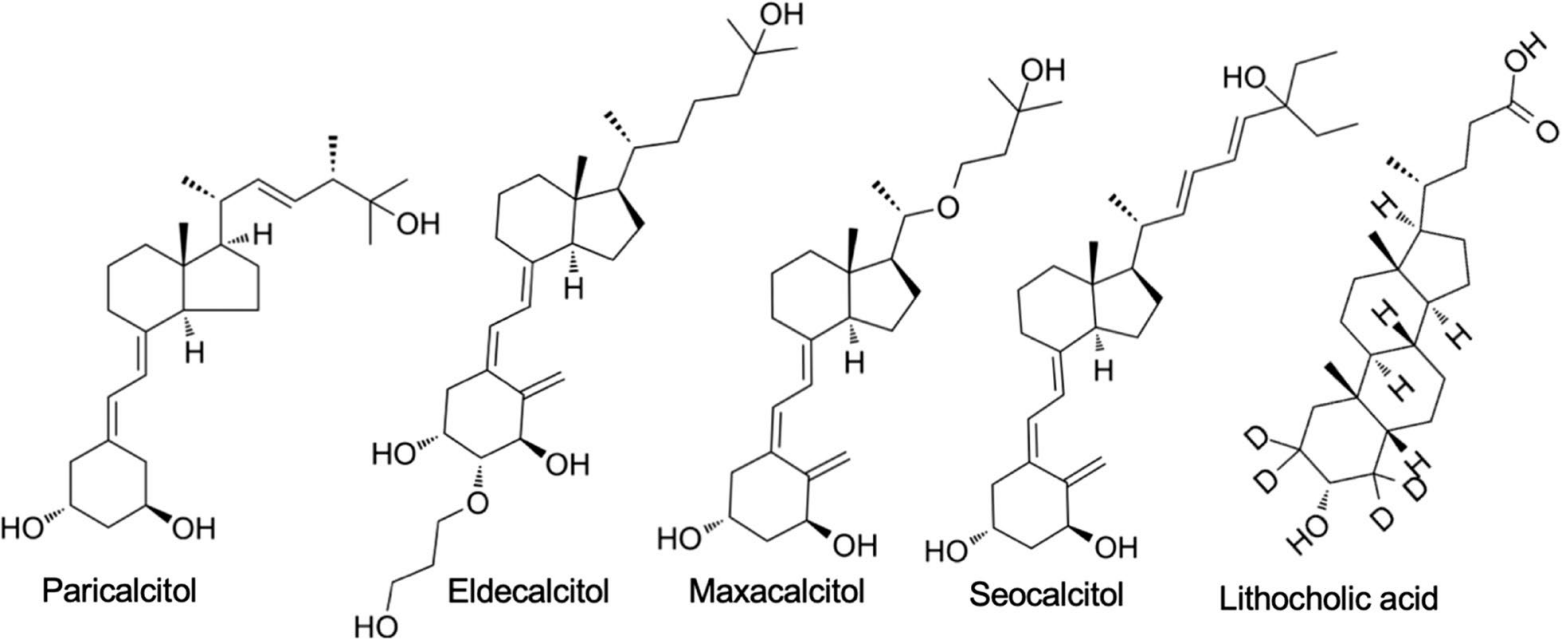
Chemical structures of VDRMs. Chemical structures of dfferent VDRMs, such as paricalcitol, eldecalcitol, maxacalcitol, seocalcitol, and lithocholic acid, are shown

Selective vitamin D receptor modulators (VDRMs) (e.g., paricalcitol, eldecalcitol, maxacalcitol, seocalcitol, and lithocholic acid, Fig. 5) are a class of drugs that target the VDR pathway [92, 93]. These compounds interact with the VDR and selectively modulate its activity, offering the potential to enhance bone health without the side effects associated with systemic VD [92, 93].

VDRMs have shown promise in preclinical and clinical studies for their ability to promote bone formation, reduce bone resorption, and improve BMD [94, 95]. These compounds exhibit tissue-selective effects, acting specifically on bone and reducing the risk of hypercalcemia and other adverse effects associated with high-dose VD supplementation [94–96]. However, further research is needed to evaluate their long-term efficacy, safety, and potential benefits compared to traditional VD supplementation.

### Combination therapies targeting VDR pathway

Combination therapies that target the VDR pathway have gained attention as potential approaches to optimize bone health and reduce fracture risk in osteoporotic individuals. These therapies involve combining VD supplementation or VDRMs with other agents that enhance bone formation or inhibit bone resorption. For example, combining vitamin K, VD supplementation with calcium and bisphosphonates, which inhibit bone resorption, has been shown to have synergistic effects in improving BMD and reducing fracture risk [97, 98]. Other agents, such as denosumab (a monoclonal antibody targeting bone resorption) [99], teriparatide (a parathyroid hormone analog promoting bone formation) [100], and selective estrogen receptor modulators (SERMs) [101], can also be used in combination with VD-based therapies to optimize bone health.

The rationale behind combination therapies is to target multiple aspects of bone metabolism, including calcium absorption, bone formation, and bone resorption, to achieve a comprehensive and synergistic effect on bone health. However, further research is needed to determine the optimal combinations, dosages, and treatment durations, as well as to assess the long-term safety and efficacy of these approaches.

### Discrepancies in findings: VD supplementation and fracture risk reduction

In recent years, randomized controlled trials in the general population examining the effects of supplemental VD on fracture outcomes have yielded inconsistent results. Some studies have demonstrated benefits, whereas others showed no significant effects [102–107]. Several factors might account for these inconsistent findings, including the use of bolus dosing, limited sample sizes in some studies, co-administration of calcium, and baseline VD levels [102–107]. A critical aspect yet to be fully addressed is the potential variability in VDR functionality among the participants. To more comprehensively understand the correlation between VD supplementation and fractures, forthcoming experimental designs should address certain limitations. These encompass the heterogeneity of study populations, ambiguities related to the optimal blood concentration of VD, the unavailability of data on participants’ baseline VD levels, and the absence of insights into genetic variations in the VDR gene.

## Conclusion and future perspectives

In this review, we have explored the role of the VD/VDR system in the pathogenesis of osteoporosis. The VDR is a key regulator of bone metabolism, mediating the effects of VD on calcium absorption, bone mineralization, and bone remodeling. Genetic variations in the VDR gene have been associated with osteoporosis susceptibility and BMD. Furthermore, the VDR interacts with other key players in bone metabolism, including the parathyroid hormone, the calcium-sensing receptor, and the estrogen receptor, contributing to the complex regulation of bone homeostasis.

Therapeutically, targeting the VDR pathway has shown promise in the management of osteoporosis [90–93]. VD supplementation, particularly in combination with calcium, has been widely used to improve bone health and reduce fracture risk. Selective VDR modulators offer a more targeted approach, selectively modulating VDR activity to enhance bone formation and reduce bone resorption [90–93]. Combination therapies that combine VDR-targeting agents with other bone-modifying drugs have also shown potential synergistic effects [97, 98].

Despite significant advancements in our understanding of the VDR and its role in osteoporosis, several areas warrant further investigation. First, more studies are needed to elucidate the mechanisms underlying the interplay between the VDR and other key players in bone metabolism, such as PTH, CaSR, and ER. Understanding these interactions at a molecular level may uncover novel therapeutic targets for osteoporosis. Furthermore, the influence of genetic variations in the VDR gene on osteoporosis susceptibility and treatment response requires further exploration. Large-scale genetic studies, including genome-wide association studies and functional analyses, can provide insights into the specific VDR polymorphisms and their implications for personalized medicine in osteoporosis. In terms of therapeutics, future research should focus on optimizing the use of VDR-targeting agents, including selective VDR modulators and combination therapies. This includes identifying the most effective combinations, determining optimal dosages and treatment durations, and assessing long-term safety and efficacy in diverse patient populations. Additionally, the potential role of VDR-based therapies in other bone-related conditions, such as osteomalacia and secondary osteoporosis, should be explored. Lastly, the integration of emerging technologies, such as omics approaches and advanced imaging techniques, can enhance our understanding of the VDR pathway and its interactions with other molecular networks in bone metabolism. These technologies can provide a comprehensive and multidimensional view of the molecular mechanisms underlying osteoporosis, paving the way for personalized and precision medicine approaches.

In conclusion, the VDR pathway plays a critical role in the pathogenesis of osteoporosis, and targeting this pathway holds significant therapeutic potential. Continued research efforts are needed to unravel the complexities of the VDR network, explore genetic influences, optimize therapeutic strategies, and leverage emerging technologies. These advancements will contribute to the development of novel treatments and improve outcomes for individuals with osteoporosis in the future.

## Data Availability

The data underlying the present study are available on request (corresponding author).
